# Integrating general practitioners into crisis management would accelerate the transition from victim to effective professional: Qualitative analyses of a terrorist attack and catastrophic flooding

**DOI:** 10.1080/13814788.2022.2072826

**Published:** 2022-05-27

**Authors:** Bernard Clary, Bélinda Baert, Gérard Bourrel, Michel Amouyal, Béatrice Lognos, Agnès Oude-Engberink, Elodie Million

**Affiliations:** a Desbrest Institute of Epidemiology and Public Health (IDESP), Montpellier University, INSERM, Montpellier, France; b Department of General Practice, Montpellier University, Montpellier, France; c University La Source Multiprofessional Health Centre, Vergèze, France; d University Pauline Lautaud Multiprofessional Health Centre, Saint Georges d'Orques, France; e University Avicenne Multiprofessional Health Centre, Cabestany, France

**Keywords:** Qualitative designs and methods, general practice/family medicine, general, disasters, terrorist attacks, flooding, general practitioner, PTSD

## Abstract

**Background:**

In 2018, Trèbes, 6,000 inhabitants with nine general practitioners (GPs) in southern France, experienced two tragedies; a terrorist attack in March, in which four people were killed, and a catastrophic flood in October, in which six people died and thousands more were affected.

**Objectives:**

We aimed to obtain a substantive theory for improving crisis management by understanding the personal and professional effects of the two successive disasters on GPs in the same village.

**Methods:**

This qualitative study conducted complete interviews with eight GPs individually, with subsequent analyses involving the conceptualisation of categories based on grounded theory.

**Results:**

The analysis revealed that GPs underwent a double status transition. First, doctors who experienced the same emotional shock as the population became victims; their usual professional relationship changed from empathy to sympathy. The helplessness they felt was amplified by the lack of demand from the state to participate in the first emergency measures; consequently, they lost their professional status. In a second phase, GPs regained their values and skills and acquired new ones, thus regaining their status as competent professionals. In this context, the participants proposed integrating a coordinated crisis management system and the systematic development of peer support.

**Conclusion:**

We obtained valuable information on the stages of trauma experienced by GPs, allowing a better understanding of the effects on personal/professional status. Thus, the inclusion of GPs in adaptive crisis management plans would limit the effects of traumatic dissociation while increasing their professional effectiveness.

KEY MESSAGES

- GPs living during major crises experienced a double status transition between healthcare professionals and victims.
- Integrating GPs into crisis management would accelerate the transition from victim to effective professional.
- GPs demanded an immediate integration into the emergency system to provide victims with the benefit of their skills.

## Introduction

Attacks and floods are disasters that impact people’s lives worldwide; they have become increasingly common in Europe [1,2]. Many terrorist attacks have occurred in Europe over the past six years. We can cite a series of terrorist attacks in Paris in 2015, Berlin Christmas market in 2016, the van which drove into pedestrians in Barcelona or the young suicide bomber who blew himself up at the Manchester Arena in 2017. These attacks have caused nearly 13 to 130 deaths and approximately 20 to 50 injured people. In 2021, floods in Western Europe killed more than 200 people and destroyed many buildings and infrastructure in Germany, Belgium, Luxembourg, and the Netherlands [2]. General practitioners (GPs) are also confronted with these crises on a personal and professional level, both in the context of early acute stress and longer-term problems such as posttraumatic stress disorder (PTSD) [3,4].

The town of Trèbes has a population of about 6,000 and nine GPs. It is located in the south of France in the Occitanie, halfway between the metropolises of Montpellier and Toulouse. On 23 March 2018, a terrorist attack on Trèbes resulted in the death of 4 people and 15 injured survivors. Later that year, on 15 and 16 October, Trèbes suffered a flood resulting in the deaths of 6 people. Thousands of other people were affected by the damage. On the ground, GPs found that many people had developed PTSD as predicted in the literature [4]. Many studies have examined the impact of terrorist crises on law enforcement and medical personnel [5,6]; the officials often develop anxiety symptoms or PSTD [7,8]. However, there was only one study on the experiences of nurses and GPs during the series of earthquakes in New Zealand in 2010–2011 [9]. Therefore, it seemed particularly relevant to explore the experience of the GPs in Trèbes during this succession of crises in a European population and health care system. We aimed to obtain a substantive theory for improving crisis management by understanding the personal and professional effects of the two successive disasters on GPs in the same village.

## Methods

### Design

We adopted a grounded theory approach (GT) [10] and conducted a constant comparison procedure to create conceptual categories [11]. The objective of grounded theory is to produce a theory from systematically collected facts in a logic of discovery [12].

### Theoretical orientation

We chose GT with the idea of producing a substantive, 'middle range' theory rather than a formal theory more suited to sociological research. In our experience of studying phenomena in health, the aim is rather to ‘understand how actors live a particular phenomenon in its context’ (care) by exploring their lived experience and not to produce a formal sociological theory. We use an approach with an existential-phenomenological sensibility, leading researchers to produce ‘substantive’ or ‘local’ theories. [13].

### Context

In France, victims’ care in the event of a disaster is provided by the public sector: the fire brigade and the emergency medical services of a hospital. The prefectural (local political government) crisis unit coordinates them. The GPs, who are liberal (private medicine), have no defined place in this institutional organisation [14]. In Trèbes, GPs work, as in the whole of France, in private practices, alone or in a group, providing primary health care and prevention to the population.

### Participants

To recruit participants, we contacted by telephone the nine GPs practising in Trèbes in 2018, between two and twelve months after the terrorist attacks and the catastrophic flooding.

We conducted purposeful sampling. The selection of participants was exhaustive and there were no exclusion criteria because we wanted to explore the experience of all doctors in the area affected by these disasters. These doctors (four women and five men) were treating the concerned population either alone (1) or in a group (8). Only one of the nine doctors refused because it was too painful for him (Table 1).

**Table 1.** Description of participants.

Sample	Gender	Age (years)	Years of practice as a gp	Years of practice in trèbes	Duration of the interview (minutes)
E1	F	54	27	27	34
E2	F	60	30	16	10
E3	M	60	34	34	30
E4	M	37	5	4	51
E5	F	37	6	6	19
E6	M	60	30	30	63
E7	F	37	8	8	40
E8	M	58	30	4	30

### Data collection

The principal investigator was a final year medical student present during the floods for supervised autonomy placement. She conducted individual semi-structured phenomenological interviews as proposed by Glaser and Strauss [10,13]. Participants were interviewed at times convenient to them and in their respective offices. The subject of the study was introduced at the beginning of each session. Socio-demographic information for each participant was collected *via* a self-administered questionnaire. To establish consistency, the main researcher and the second researcher, developed the interview guide below. This guide was developed from international literature data, containing open-ended questions focussing on the GPs' lived experiences, thus appealing to their memories and reflexivity in all dimensions (psychological, behavioural and emotional) [15]. Data were collected from December 2018 to October 2019. The interviews lasted an average of 35 min each.

Where answers required clarification, follow-up questions were sent to help participants elaborate their thoughts [15]. A pilot test was not conducted due to the completeness of the sample. The principal researcher recorded and then transcribed each interview in its entirety using Microsoft Word^@^ 2010. Moreover, non-verbal communication (e.g. attitude, mimicry, onomatopoeia) was indicated *via* italicised square brackets, while silences were represented with suspension points; words expressed with emphasis were written in uppercase (Table 2).

**Table 2.** Interview guide.

Remember the day of 23 March 2018 What happened? Can you tell me about this day? What was your experience? In the aftermath of this day, what has been the impact on your practice? Do you remember any particular facts or situations related to this event that changed your daily practice? What did you feel or do at the time? In the months that followed, what was your emotional and psychological state? Have there been any changes in your professional life or activities? Today is 15 October 2018. The commune of Trèbes is once again facing an exceptional event less than seven months after the attacks, with deadly floods that have claimed 1,200 victims in the commune. How did you feel at that time? What changes have you noticed in your daily working life? Are there elements or aspects of these two experiences that could be useful to your practice in crisis situations, or in similar situations? How have these events changed your vision of the future? Our interview is almost over. Is there anything else you would like to add?

### Data analysis

We used the Grounded Theory method inspired by Glaser and Strauss [10]. The analyses were performed simultaneously as the interviews, enriching our questions to deepen a particular theme or the understanding of a category per the other principles of theoretical sampling. [16]. We did not choose concepts a priori, using an emergent discovery logic. We divided the text into units of meaning, which we annotated as open coding. Axial coding, line by line and transversal coding (by constant comparison with other verbatims) allowed us to establish themes and their properties to identify a category. The category is constructed by taking the analysis to a conceptual level by assembling themes relevant to the research question. At this stage, the categories can be reduced to incorporate a more conceptually abstract concept of which they are properties or sub-categories. The process of constant comparison increases the generality of the categories until they are saturated, when no more new information is added (selective coding). Each category is a dimension of the phenomenon studied. The synthetic restitution leads to a main category in the form of a general integrative proposition from the different information contained in the individual categories and their properties. Theoretical saturation was reached in the sixth interview. No new themes were identified. There were no negative cases that could have disproved the emerging theory.

### Trustworthiness

Trustworthiness was ensured by the rigour of the constructive procedures and the triangulation of the data [17]. Consistency and validity were achieved by matching the choice of methodological approach to the research question. The second researcher, GP and academic researcher, conducted a cross-analysis of all interview data (triangulation), ‘to keep the inquirer objective’ [18]. Reliability, constant comparison, and saturation make it possible to envisage the transferability of results in similar situations, territories, and health systems. To write this study, we followed the Consolidated criteria for reporting qualitative research (COREQ) [19].

### Ethics

Each participant signed an informed consent form beforehand. All participants were given the option to receive full transcripts of their respective interviews by mail, which they declined.

The protocol was approved by the University Department of General Medicine of Montpellier (Montpellier, France). Approval by an Ethics Committee was not needed because of this work’s non-pharmaceutical biomedical research nature (French Jarde law). Data anonymisation was immediate, as each participant was identified with a unique code (e.g. E1, E2). Any name mentioned by a participant was recorded in the transcript as is. Further, the names of people or places were either deleted or anonymised.

## Results

Four categories emerged from the analysis to understand the personal and professional effects of the two successive disasters on GPs.

### Category 1

GPs initially perceived themselves as victims. They immediately felt an emotional shock paralysing their thinking and actions. They felt a heightened sense of belonging to the community by sharing the same suffering as the inhabitants. Their emotional shock was amplified by the memory of previous disasters, the number of people affected, repeated disasters and the intense media coverage. The process of identifying the victims transitioned the professional relationship moved from empathy to sympathy.

The GPs suffered brutal violence and emotional shock like all the inhabitants of Trèbes. Then, they experienced an immediate sense of powerlessness and vulnerability that may have paralysed their thinking and action.

*There is an emotional climate present everywhere, everywhere, everywhere, everywhere! And so, you are in it! You are in the vortex. […] What was extremely violent was the thousands of people, well the thousand people… who found themselves losing everything! Losing everything…. [tears]. [E3]*

The need to continue an everyday life as a citizen or leave was a source of guilt. Some GPs tried to lead a ‘normal’ life detaching from the events, which may have led to some guilt. They felt it was necessary to move away from Trèbes to limit their emotional exhaustion.

*Getting involved in cleaning up the city required a complicated schedule. Therefore, we stuck to our schedule! We preferred to get out of the situation to do something else rather than talking about it all the time! [E7]*

The professional doctor-patient relationship has moved from empathy to sympathy. GPs identified with the victims, which changed their professional relationships. They expressed an increased sense of community and a sense of purpose in sharing the same suffering. Initially, sympathy dominated the doctor-patient relationship. However, some participants spoke of compassion fatigue during the second disaster.

*I feel more effectively a member of the community of the people of Trèbes. That's for sure… than before! [E6]*

Exogenous factors have amplified violent experiences. These events brought old traumas to the surface (note that one doctor refused to participate in this study). The number of people affected and the repetitive traumas amplified the emotional shock. The impact of the floods was greater because the terrorist attack had sensitised the GPs.

*So there I felt… first of all… really the impression that it's happening again because I lived through the 1999 floods in the Aude. [E8]*

*… it was more painful at the time! Because it was the second shock. [E5]*

Furthermore, the psychological impact on GPs was amplified by frequent and intense media coverage and the presence of national political figures in a commune that had already suffered a strong stigma.

*The Prime Minister came, there was a funeral for the… the… the victims… with an emotional and media process that was… huge! [E3]*

### Category 2

The lack of integration of GPs into the crisis management system has led to feelings of frustration, abandonment, anger, lack of recognition and powerlessness.

GPs felt frustrated by the lack of demand and recognition from public authorities. They wondered about their position in the organisation of healthcare in this exceptional situation and thus felt abandoned.

They felt anger towards the institutions that had neglected them at a regional level. Some participants suffered from the state’s lack of recognition and indifference, even though they had done their duty.

*And then the absolute indifference of the authorities towards general medicine in a disaster situation. With an … under-use … and ignorance of all the work we have done […] And so, as we are not in the plans, we are nobody! [E6]*

### Category 3

After a period of immediate shock, the professional relationship of GPs that had been transformed into sympathy became empathetic again in a second statutory transition. They recovered their professional values and skills and acquired new ones. They demonstrated reflexivity in mobilising their skills of holistic approach to patients, empathy, follow-up consultations, and coordination of care. They also acquired new skills and demonstrated adaptation, a resilience factor.

GPs generally demonstrated reflexivity in mobilising their skills with a holistic approach to patients, empathy, and follow-up consultations and care coordination. However, some needed to compose themselves.

*… I needed to settle down, to be a human being and not a war machine and…! [E5].*

GPs have acquired new skills and shown adaptation and resilience. They developed new skills to adapt to crises, which they then integrated into their usual practices. GPs had to adapt their daily practices during the first few weeks of the disaster. For some GPs, the long-term effects on their medical practice were not important because they were used to adapting. Even if GPs describe the events as an enriching experience, they regret the difficulty of working with limited resources and infrastructure, particularly to a wartime medicine that was not their own. However, GPs have shown resilience by rediscovering the pleasure of being helpful, enhancing their skillset and giving hope.

*And we are lucky enough to pass all this on to people because, in the end, the meaning of life is to be useful to our children, our friends, our patients! Give them the desire, give them hope. [E1]*

### Category 4

GPs, concerned that their population would be affected, wished to be included in a coordinated crisis management system. They would have liked to have contact with psychological support cells during disasters. They valued peer groups as psychological support for themselves.

Participants felt that GPs should have been included in a coordinated crisis management system for their catchment areas, including public institutions and private practices. GPs would have liked to be coordinated with the psychological support cell.

*Better coordination between us, the liberals, and… the public bodies that deal with all this [….]. There is a bit more work to do in coordination with the psychological emergency cell: where we have no contact. [E8]*

None of the participants said that they had personally consulted a PTSD professional, although some would have liked the opportunity to join a focus group to share their experiences. To limit emotional exhaustion, peer support was seen as beneficial, both in the direct experience and in the recovery process.

*I think we are lucky to be in a place where there has been a lot of solidarity between doctors. [E3]*

*And so that made me feel outstanding! To hear my friend, say, 'Well, we'll do the impossible so you can transfer them’ [the elderly who were in the flooded nursing home], we'll take care of it. [E6]*

### Substantive theory

The analysis revealed that GPs had undergone a double status transition.

First, GPs had undergone the same emotional shock as the population with a sense of belonging to the community of victims. This emotional shock changed the usual professional relationship from empathy to sympathy. The transformation temporarily caused a shift from the status of health professionals to that of victims (first status transition). The feeling of powerlessness and loss of legitimacy was amplified by the absence of a request from the state to participate in primary emergency measures, which caused them to lose their professional status.

In a second phase, GPs recovered these professional values and skills and acquired new ones to care for their patients. This is the moment of return to their status as competent professionals with adaptation skills and of resilience (second statutory transition).

In this crisis period, participants proposed the integration of GPs in a coordinated crisis management system and the systematic development of peer support.

## Discussion

### Main findings

We aimed to understand the personal and professional effects of the two successive disasters (terrorist attack and major flood) on GPs in the Trèbes village.

The analysis revealed a substantive theory. GPs have undergone a double status transition between healthcare professional status and victim status before returning to the professional position. First, GPs had undergone the same emotional shock as the population changing the usual professional relationship from empathy to sympathy. It caused a shift from the status of health professionals to that of victims (first status transition). The victim status deals with a feeling of powerlessness and loss of legitimacy amplified by the absence of a request from the state to participate in primary emergency measures. In a second phase, GPs recovered these professional values and skills and acquired new ones to care for their patients. They returned to their status as competent professionals with adaptation skills and resilience (second statutory transition). In this crisis period, participants proposed the integration of GPs in a coordinated crisis management system and the systematic development of peer support.

#### Comparison with the literature

##### Double status transition

The conceptual categories that emerged from the verbatims suggest a substantial theory of double status transition [20]. We did not find similar results in the literature. This substantive theory of double status transition can enrich the formal theory of Glaser and Strauss about the statutory transition [21]. Different propriety of this formal theory can be found in our substantial theory. We can find the form propriety of our theory: we can say it is like a circle between the two statuses. The temporality is found in the two different stages between the two statuses and the time staying in each status, which can differ for each GP. Of course, we can discuss the desirability: the victim status was probably less desirable than the competent health care professional for GPs but we can ask ourselves if the transition to victim status had a role in the increase in professional skills that GPs felt when they returned to professional healthcare status.

##### Traumatic dissociation or tonic immobility

Our findings highlight those participants experiencing an initial emotional shock, which created barriers to their thinking and acting. French psychiatrist Muriel Salmona, who led a national survey on sexual violence in 2015, has shown that this process can also arise from terrorism and other attacks, often causing a ‘cessation of thought’ which she called ‘traumatic dissociation’ [22]. This state of involuntary and temporary motor inhibition is like ‘tonic immobility’, described as a universal process observed in traumatic shocks such as rape [20]. This tonic immobility is associated with the development of subsequent post-traumatic stress disorder and severe depression in the study by Moller *et al.* Our qualitative study could not quantify this phenomenon. Still, in the IMPACTS study of emergency and law enforcement/response health professionals who were deployed to deal with the Paris attacks and hostage-taking, 3% developed post-traumatic stress disorder (PTSD), and at least 14% developed an anxiety disorder [7]. For the 2016 Berlin terrorist attack, one study showed a slight increase in levels of aggression and hostility among police officers and significantly lower environmental quality of life among firefighters [8]. Previous studies had shown that PTSD was associated with the intensity and duration of exposure, lack of preparedness and social isolation [5].

In this context, the participants felt that they were ‘inside’ a community of destiny and, therefore, experienced a sense of community shock. They immediately identified with the victims. For Gershuny and Thayer, social loss is a phenomenon felt by all [23]. However, Johal and Mounsey did not report this in the New Zealand earthquakes [9].

##### Adaptation and resilience

In our study, GPs underwent a dynamic status transition in which their status shifted from healthcare professional outside the population to being a victim and then back to being competent professionals in the community. This double transition status has had professional and personal impact on GPs. Literature is rich about such effects on healthcare professionals experiencing such catastrophic crises: GPs have acquired new adaptive skills (active listening, empathy) [24], through immersion in the local community, while fostering resilience through the ‘pleasure of being useful’, peer solidarity and social support [5]. Consultations have become longer due to the lack of psychotherapeutic referrals, and GPs have noted an increase in the use of care for somatic disorders [7,9,25].

##### Proposals for improvement

A clear place should be defined for GPs in crisis management plans, thus avoiding circumstances in which they are equated with victims.

To date, there are few primary care recommendations for the management of victims of terrorist attacks or natural disasters [26]. Most studies exploring these situations have focussed on emergency/response services rather than on the experiences of 'local' health professionals such as GPs. In France, the crisis plan of the Directorate General of Health specifies the important role that GPs can play in situations of collective aggression by weapons of war, including attacks but does not address the type of support they may need [14].

Over the last 10 years, significant progress was achieved concerning both the knowledge and management of PTSD, including genomics, neurobiology, links between forms of trauma exposure and their lifetime effects, early interventions, improved psychological/medicinal treatments, and synergistic approaches. There have also been essential research perspectives on social support, sex, gender, and resilience [27].

Our results also showed that coping skills, peer support and sharing experiences were essential for GPs' resilience and health. These results are consistent with those of Skryabina and all, about qualitative interview data from frontline staff who responded to a prominent mass casualty terrorist incident in the UK in 2017. They found that social support provided by peers and organisational debriefs were identified as two most common support mechanisms after psychological trauma were both essential elements in building resilience and, therefore, professionally effective [28].

### Strengths and limitations

Our study is highly original in the context of European GPs experiencing a double catastrophic crisis. While there is a large body of work on PTSD and its management in civilian populations [29], we found only one study in the literature examining the experiences of nurses and general practitioners in a similar type of crisis, namely the 2010–2011 earthquake series in Canterbury, New Zealand [9].

We conducted a targeted exhaustive interview study of Trèbes physicians practising during the attack and flooding. This choice could be a limitation as it would not seem to correspond to the criteria of a classic theoretical sampling. However, it is adequate for our research because it takes place in a French village with only nine GPs and, as said by Bowen in his 2008 article: ‘it is not always necessary to increase the sample size [17]. We also have not interviewed other GPs who had experienced other disasters in Europe because it was not the purpose of our study. It could have affected the theoretical saturation and transferability of our substantive theory. However, the data collection and the analysis carried out simultaneously allowed to deepen a particular theme or the understanding of a category per the principle of theoretical sampling. The theoretical saturation was reached in the sixth interview and the last two interviews do not add new themes or negative cases. About the transferability, we believe our substantive theory is transferable to similar care systems where GPs are not initially integrated into collective emergencies. Our theory is a substantive; it would be necessary to have other substantive theories to analyse in another context to reach a fundamental formal theory.

The principal investigator was a final year medical student. She knew the physicians interviewed and experienced the events; this may have influenced the authenticity of the responses and the validity of the analysis. To limit a possible bias, investigators gave individual attention, with careful supervision, to the GPs interviewed.

### Practical implications and prospects

In this study, we highlighted the lived experience of GPs who worked during two traumatic health crises. This resulted in a double status transition in which participants moved from being professionals to victims to competent professionals, creating tension between their professional and personal experiences. They also reported feelings of frustration and illegitimacy, as they were not included in the crisis mechanism. The understanding of this double statutory transition of the GP during crises is vital to consider by institutions and professionals. This is to optimise the role and facilitate the experience of each health professional involved in the management of these health crises.

## Conclusion

GPs experiencing major crises may experience status transitions between professional and victim. These transitions can be amplified by an undefined place in the organisation of the rescue during these events. In the future, and in all countries, it will be essential to establish a clear position for GPs in the crisis management system, thus showing an interest in their health while increasing their professional effectiveness. In the context of recurrent crises, which are likely to occur during generalised disasters, future studies should examine the roles of primary care professionals in working within adaptive crisis management systems.

## Data Availability

All interviews are available at the Department of General Medicine, University of Montpellier, Montpellier, France.
